# Unexpected sequel to the application of silver fluoride followed by stannous fluoride to an open carious lesion in a primary molar: A case report

**DOI:** 10.1002/cre2.838

**Published:** 2024-03-20

**Authors:** Graham G. Craig, Jeffrey X. Shi

**Affiliations:** ^1^ Dental Outlook Camperdown New South Wales Australia; ^2^ School of Chemical and Biomolecular Engineering The University of Sydney Sydney New South Wales Australia

**Keywords:** caries surface, silver fluoride

## Abstract

**Objectives:**

The use of silver fluoride followed by stannous fluoride was designed for the treatment of open carious lesions in primary molars in dental outreach programs. However, during the COVID‐19 pandemic when aerosol‐producing procedures were inadvisable, one dental location started using it as the first stage in a two‐visit restorative procedure for carious primary molars. If the gap between the fluoride application and the restoration placement stages was around 3–5 weeks it was noticed that a black friable crust appeared on the caries surface. To investigate further a normally discarded crust from one patient was retrieved and sent for analysis.

**Materials and Methods:**

Two techniques suitable for identification and preliminary analysis of material of unknown composition, scanning electron microscopy and energy dispersive spectroscopy (EDS) were used. The only preparation was that the specimen was dried and coated beforehand.

**Results and Conclusions:**

This preliminary examination showed two unexpected findings. The first was that the crust surface indicated a possible dentine derivation as it was covered with reasonably evenly spaced holes. In addition, the EDS spectrum showed it to be, at least, partially mineralized. The second unexpected finding was that the surface was coated with electron‐dense particles. The size of the particles and the EDS spectrum pointed to the likelihood of the majority of them being nanosilver. These unexpected findings suggest a possible new direction for research.

## 1 INTRODUCTION

A two‐step topical application procedure for active carious lesions in primary molars involving the use of silver fluoride followed by stannous fluoride immediately turns a lesion black. Retention of the black stain is indicative that the underlying lesion has not progressed, whereas loss of the stain is a sign of lesion progression (Craig et al., 2013). This aspect is useful for diagnostic purposes, particularly in dental outreach programs.

During the COVID‐19 pandemic when nonaerosol‐producing techniques were indicated, one dental facility used the above approach as an initial step for carious primary molars. This was followed at a subsequent visit by the placement of a glass‐ionomer cement restoration. In essence, it followed the delayed restoration‐placement protocol with the Silver Modified Atraumatic Restorative Technique, which uses silver diamine fluoride (SDF) as the initial treatment (Natarajan, 2022).

During this period, an unexpected phenomenon was noted. If the gap between initial treatment and restoration placement was around 3–5 weeks, a black, friable crust was seen on the caries' surface. It resembled burnt toast. Normally, before restoration placement, it was lifted off and discarded.

To investigate this situation further, a retrieved discarded crust from one patient was submitted for preliminary analysis. It was of particular interest because previously the effect of silver fluoride followed by stannous fluoride on active carious lesions in primary molars had been made at baseline and after 6 months (Craig et al., 2013). Any in‐between situation such as the one observed would not have been noted or its possible significance investigated.

## 2 CASE DESCRIPTION AND RESULTS

An open carious lesion on the occlusal surface of a lower primary second molar in a 6‐year‐old boy was treated with aqueous 40% silver fluoride followed 3 min later by an application of 10% stannous fluoride (Caries Status Disclosing Solution; Creighton Dental). To temporarily exclude saliva, the site was covered with a fluoride varnish.

Three weeks later when the patient returned to have a glass‐ionomer cement restoration placed a black surface crust was seen on the formerly active caries surface. It was scraped off and the discarded crust was retrieved. It was placed in a 0.2 mL polymerase chain reaction vial with a domed cap and, with signed parental approval, forwarded for analysis. No attempt was made to add any form of preservative or transport medium.

It was accompanied by an ethical approval statement and a free and informed signed parental consent form.

On receipt, as it was an initial exploration, it was decided to examine the sample using scanning electron microscopy (SEM) and energy dispersive spectroscopy (EDS). The specimen was dried, mounted, and a conductive coating placed before being examined using SEM with a Zeiss Sigma Field Emission SEM (Carl Zeiss Microscopy) and a Phenom X Desktop SEM (Thermo Scientific). The latter was operated at an acceleration voltage of 15 kV, which would have given an electron beam of just over 1 µm in diameter. Both instruments had energy dispersive spectrum facilities.

**Figure 1:**
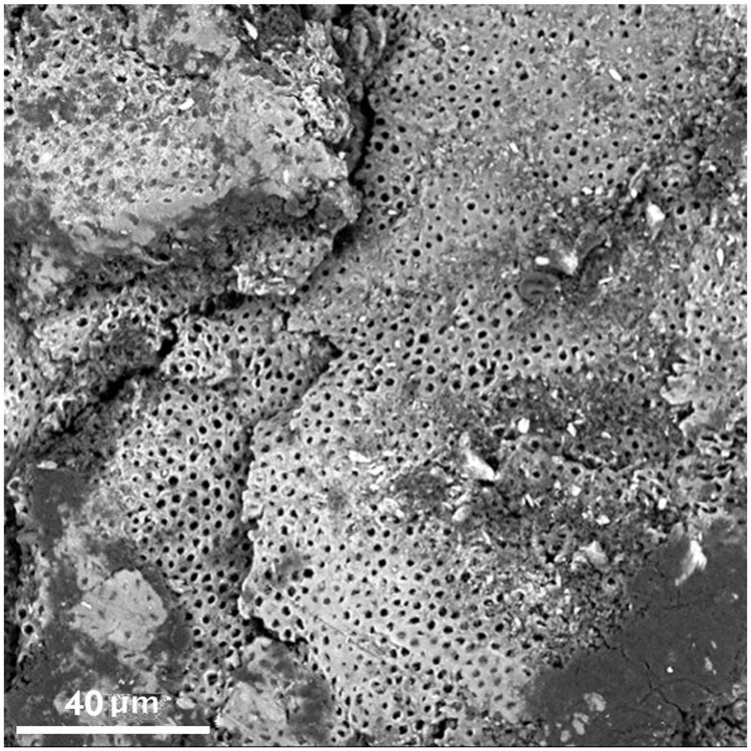
Low resolution scanning electron microscopy view of the discarded caries surface crust showing relatively evenly spaced holes suggestive of a dentine origin. There is no evidence of bacteria, bacterial remnants, or organic deposits.

**Figure 2:**
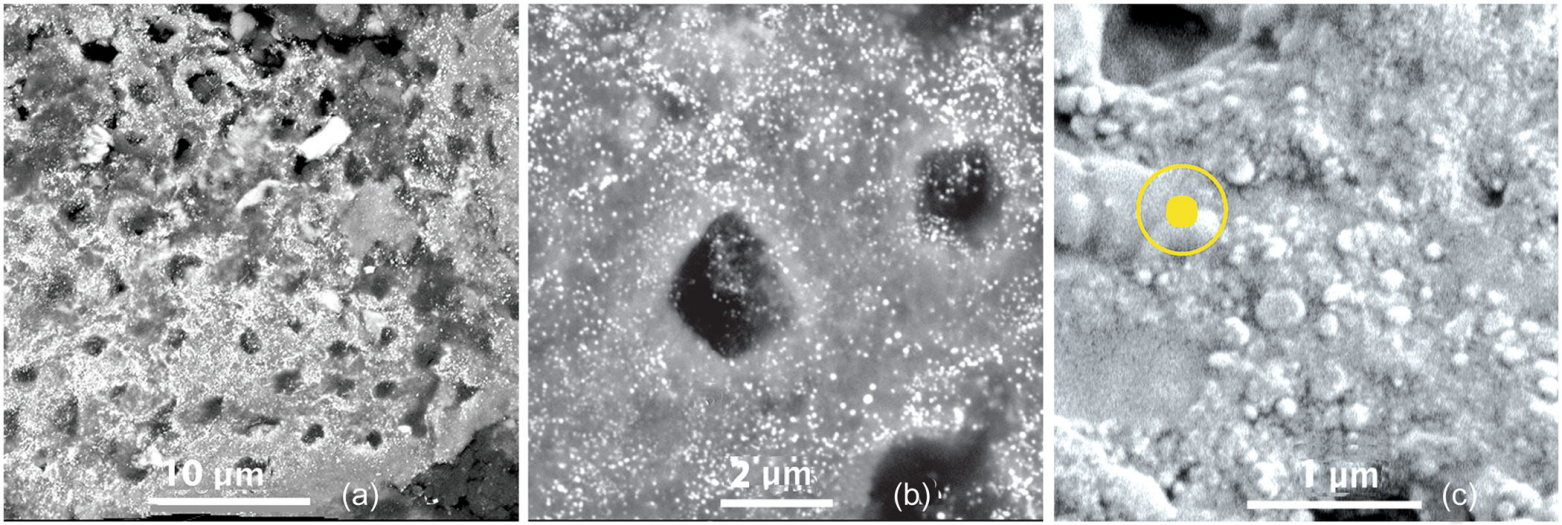
Increasingly higher resolution scanning electron microscopy views showing electron‐dense particles widely distributed over the discarded crust surface (a) with no obvious clumping (b). The highest resolution view (c) with a 100 nm dot circled shows the majority were this diameter or less.

**Figure 3:**
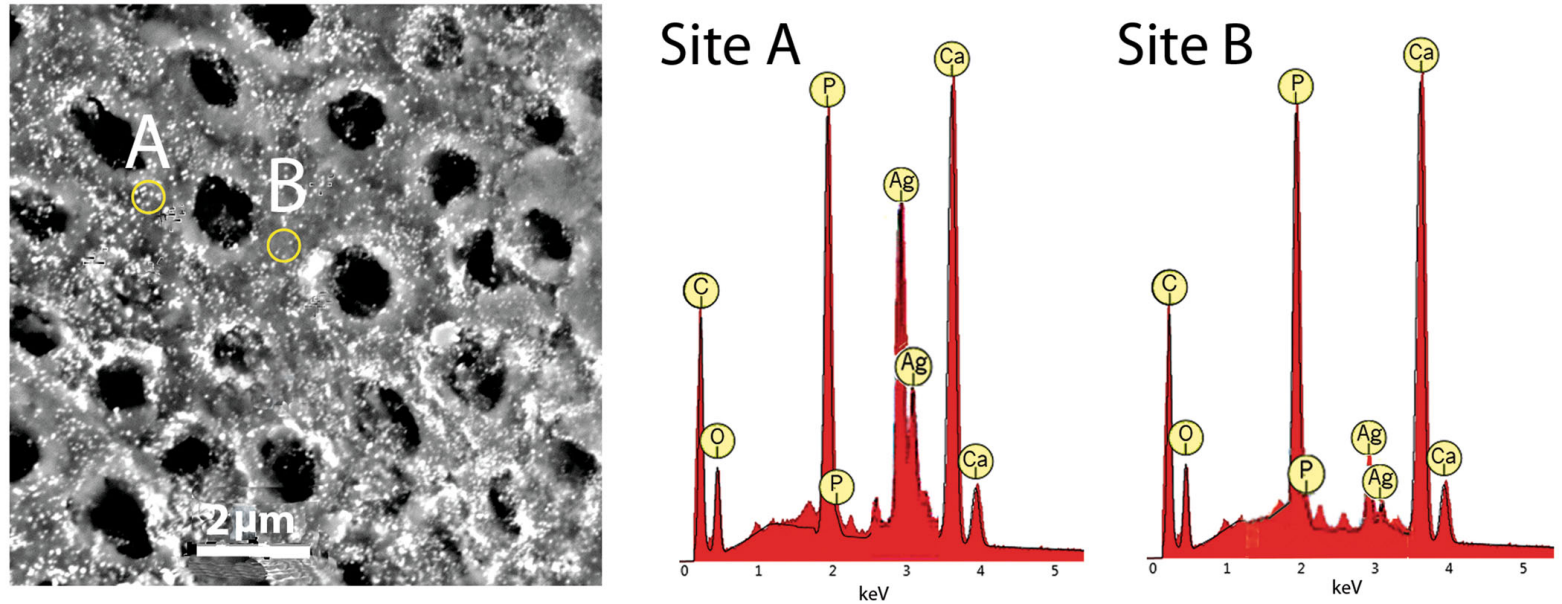
Energy dispersive spectroscopy for two spot sites selected at random. The circles show the estimated area covered by the electron beam. Both sites have high peak heights for calcium and phosphorus and less for oxygen. Site A has a higher concentration of dense particles than Site B and has more pronounced peak heights for silver.

The low‐resolution SEM view showed a surface with relatively evenly spaced small holes with an absence of bacteria, bacterial remnants, or organic debris (Figure 1). Higher resolution views showed small dense particles covering the entire surface, the majority of which were around 100 nm or less in diameter (Figure 2). Because the surface was not flat or polished and contained porosities it was not feasible to carry out an accurate quantitative EDS analysis. The EDS data for spot analysis of the dense particle sites selected at random all showed marked peak heights for silver as well as calcium, phosphorus, and oxygen. In areas with no obvious dense particles, there were no appreciable peak heights for silver but were present for calcium, phosphorus, and oxygen. Examples of these observations are shown in Figure 3.

## 3 DISCUSSION

Examination of the discarded caries crust gave, in particular, two unexpected, and previously unreported, findings. The first was that the surface had relatively evenly spaced holes suggestive of a dentine framework. This provided a strong indication that it was derived from dentine beneath the, no doubt present, infected surface layer on the original active carious lesion. The absence of any evidence of bacteria or bacterial remnants suggests that the infected layer was lost in the period following the original treatment. Furthermore, the EDS data showed marked peak heights for calcium, phosphorus, and oxygen at all sites examined including ones without any obvious dense particles. This is indicative of some degree of mineralization of the discarded surface crust and whether the elements identified were from the original dentine or picked up from the patient's saliva is open to question. It has been established that SDF can inhibit the degradation of collagen (Mei, Ito, et al., 2013). So it is possible that the silver fluoride in the topical application had a similar effect and helped preserve the dentine architecture.

The second unexpected finding was to observe the specimen surface coated with small dense particles, the majority of which were 100 nm in diameter or less. EDS findings for sites with these particles gave high peaks for silver. It is noteworthy that in sites where these particles were quite sparse, there were no high peaks for silver. The particles could be nanosilver.

The presence of small silver particles following treatments with SDF has been seen previously. Very small particles of silver, which were dispersed and difficult to analyze using SEM‐EDX, were noted in arrested lesions previously treated with SDF in the primary teeth of a 6‐year‐old boy (Mei et al., 2014). Also, following an SDF application, silver particles 0.5–1.0 µm in diameter developed in vitro in dentine carious lesions created with *Streptococcus mutans* and *Lactobacillus acidophilus* biofilm (Mei, Chu, et al., 2013). A similar treatment of a plaque biofilm on enamel slabs worn by volunteers also produced silver particles of unspecified size (Klanliang et al., 2022). No reports were found of specific examinations for nanosilver.

If the particles seen in this case report were nanosilver, several factors could have contributed to their formation. Initially, the silver ions in the silver fluoride would have been reduced and the stannous ions in the stannous fluoride would perform this function. The stannous ions from stannous chloride have been used in the synthesis of AgNPs (Babaahmadi & Montazer, 2015). Two other constituents of the stannous fluoride solution, glycerol, and sorbitol, could also have played a role. Glycerol is a useful solvent for AgNPs (Liu et al., 2020). Sorbitol has been shown to help make uniform‐sized AgNPs dispersed in a medium (Steinigeweg & Schlücker, 2012). In addition, the bacteria in the carious lesion could have contributed to the bacteria‐mediated formation of AgNPs, which has been reported previously (Klaus et al., 1999).

Finally, it was unexpected that there was no discernible evidence of either tin or fluoride on the specimen surface even though both the moieties were present in the treatment solutions.

The current investigation was exploratory and intended only to provide a general overview of the sample. However, now evidence of nanosilver has been found, which provides a direction for future research using more precise tests such as transmission electron microscopy. This method is regarded as a valuable tool for the characterization of nanomaterials as it provides quantitative measures of particle size, size distribution, and morphology. However, unlike scanning electron microscopy, it requires serial sections and therefore a breaking up of the specimen (Lin et al., 2014).

Whether the unexpected observations in this report are representative of a more widespread occurrence with other silver fluoride‐based preparations, such as SDF, is not known. Nonetheless, they open up a hitherto unexplored area that warrants further investigation.

## AUTHOR CONTRIBUTIONS

Graham G. Craig received the specimen, arranged for its analysis, and wrote the manuscript. Jeffrey X. Shi carried out the SEM and EDS analysis. Both authors read and approved the manuscript.

## CONFLICT OF INTEREST STATEMENT

The authors declare no conflict of interest.

## ETHICS STATEMENT

Written informed consent was obtained from the patient's parent for publication of this case report.

## Data Availability

Patient personal record is not made available due to patient confidentiality.
